# Double-stranded microRNA mimics can induce length- and passenger strand–dependent effects in a cell type–specific manner

**DOI:** 10.1261/rna.054072.115

**Published:** 2016-02

**Authors:** Mae A. Goldgraben, Roslin Russell, Oscar M. Rueda, Carlos Caldas, Anna Git

**Affiliations:** Cancer Research UK-Cambridge Institute, University of Cambridge, Cambridge CB2 0RE, United Kingdom

**Keywords:** microRNA, siRNA, microRNA mimics, dsRNA sensing, miR-155

## Abstract

MicroRNAs are short (17–26) noncoding RNAs driving or modulating physiological and pathological cellular events. Overexpression of miR-155 is pathogenic in B-cell malignancy but was also reported in a number of solid tumors—in particular, in breast cancer, where its role remains unclear and often contradictory. Using representative cell line models, we sought to determine whether the discrepant miR-155 effects in breast cancer could be explained by the heterogeneity of the disease. The growth of six breast cancer cell lines transfected with several miRNA mimics was analyzed. We found MCF-7 cell growth to be inhibited by miR-155 and miR-145 mimics, both 23-nt long, but not by a number of shorter mimics, including a universal commercial negative control. Microarray and Western blot analyses revealed induction of apoptosis, associated with interferon-β after activation of the double-stranded RNA sensor pathway. 3′ Trimming of the miRNA mimics to 21 nt substantially reduced their growth-inhibitory potency. Mutating the canonical seed of the miR-155 mimic had no effect on the induced inhibition, which was abolished by mutating the miRNA seed of the artificial passenger strand. A panel of breast cancer cell lines showed a wide range of sensitivities to 23-mer mimics, broadly consistent with the sensitivity of the cell lines to Poly (I:C). We demonstrate two sources for nonspecific in vitro effects by miRNA mimics: duplex length and the artificial passenger strand. We highlight the danger of a universal 21-mer negative control and the importance of using matched seed mutants for reliable interpretation of phenotypes.

## INTRODUCTION

MicroRNAs (miRNAs) are a class of noncoding RNAs, generally conserved across the higher eukaryotes and typically ranging between 17 and 26 nt in length (miRBase21) (Kozomara and Griffiths-Jones 2014). Best described as post-transcriptional fine-tune modulators of gene expression, miRNAs function predominantly by directing the RNA-induced silencing complex (RISC) to the 3′ UTR of target mRNAs, leading to suppression of protein translation and destabilization of the mRNA transcript (Djuranovic et al. 2011; Meijer et al. 2013). Specific recognition of target mRNAs is mediated by imperfect base-pairing of nucleotides 2–8 of the miRNA (“seed” sequence) to the 3′ UTR of the regulated mRNA (Bartel 2009).

Canonically (for review, see Ha and Kim 2014), miRNA genes are transcribed by RNA polymerase II to yield primary miRNAs (pri-miRNAs) that go through an initial nuclear maturation stage, mediated by Drosha and its coactivator DGCR8, and resulting in imperfectly base-paired stem–loop precursors (pre-miRNAs) of ∼70 nt. After their export into the cytoplasm, a further maturation step is executed by Dicer, assisted by TRBP, PACT, and Ago2 in a poised complex, by cleaving the precursor's loop and generating a short imperfect double-stranded RNA (dsRNA) stem (miRNA duplex) (Wilson et al. 2015). Ago2 then orchestrates the last maturation step, preferentially incorporating one of the duplex RNA strands into the RISC, responsible for translational repression and RNA degradation.

MiR-155 has been extensively described for its essential role in the normal immune function of B-cell and T-cell lymphocytes and dendritic cells, but also as a likely driver in some aggressive lymphomas such as Hodgkin's and diffuse large B-cell (Vigorito et al. 2013; Seddiki et al. 2014). It has been proposed that because miR-155 is oncogenic in lympho-proliferative disorders, this may be true for other cancers. Several studies examining the expression of miRNAs in breast cancer have reported that miR-155 is associated with more invasive cancer, although closer examination shows that miR-155 expression is related to high lymphocytic infiltrate and may actually be a good prognostic indicator in more aggressive tumors (Volinia et al. 2006; Chen et al. 2012; Cascione et al. 2013; Dvinge et al. 2013).

Animal models of breast cancer and murine cell lines, albeit criticized for their relevance to human disease, have shown a cooperative interplay between miR-155 and TGF-β signaling in inducing epithelial-to-mesenchymal transition for more invasive cancer behavior, observed in MMTV-PyMT mice and NMuMG cells (Kong et al. 2008; Johansson et al. 2013). In contrast, a study of human breast cancer cell lines, ZR-75-1 and MCF-10A, showed that gain-of-function mutant p53 induces expression of miR-155, which inhibits TGF-β signaling by targeting ZNF652 to promote cancer cell invasiveness (Neilsen et al. 2013). Moreover, validated miR-155 target transcripts in the context of breast cancer include both tumor-suppressor genes, like CEBP-β, FOXO3a, ZNF652, and VHL, and oncogenes, like BACH1 and SATB1 (Yin et al. 2008; Kong et al. 2010, 2014; McInnes et al. 2012; Johansson et al. 2013; Neilsen et al. 2013).

These contradictory data regarding the pro- or antiproliferative functions of miR-155 in breast cancer prompted us to interrogate the effect of miR-155 overexpression in a panel of breast cancer cell lines representative of the breadth of breast cancer subtypes (Curtis et al. 2012; Ali et al. 2014).

Studies of individual miRNA function in model systems require perturbation of the miRNA levels. Overexpression is commonly achieved either by vectors (plasmids or lentiviruses) expressing shRNA-like miRNA precursors, which require transcription and processing by the cellular Drosha/Dicer pathway, or by delivery of artificial perfect miRNA duplexes (mimics). Similarly to their duplex siRNA counterparts, miRNA mimics do not require processing and get directly incorporated into RISC to exert their effect on target mRNAs, ultimately leading to measurable cellular phenotypes. We have found that the use of miRNA mimics has caveats that are overlooked and might become especially important as miRNAs are being proposed as therapeutic agents.

## RESULTS

**FIGURE 1.**
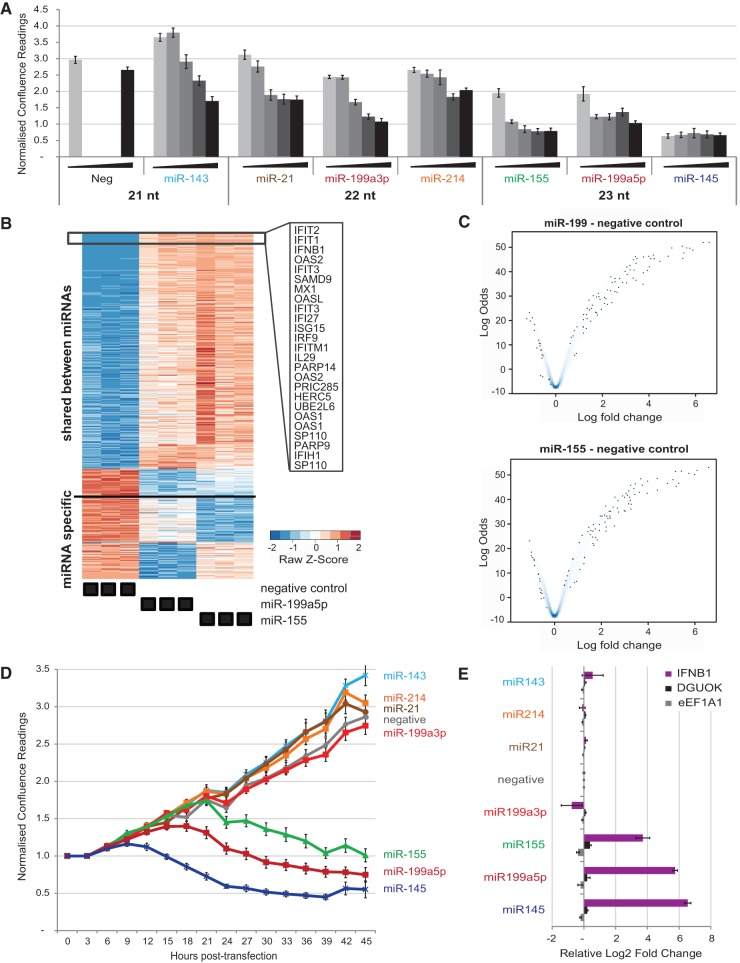
Several miRNA mimics trigger interferon response and reduce the growth of MCF-7 cultures. (*A*) Culture confluence of MCF-7 cells (normalized to initial confluence) was measured using an IncuCyte at 48 h post-transfection with increasing concentrations of miRNA mimics (5, 10, 25, 50, and 100 nM). Negative control mimic was tested only at 5 and 100 nM. Results are sorted by the lengths of the tested miRNA. Error bars, SEM of nine scans per well (*n* = 2). (*B*,*C*) Heatmap of unsupervised clustering (*B*) and LogOdds/FoldChange volcano density plots (darkness of blue shade proportional to density of probe data points) (*C*) of 1005 microarray-based differentially expressed genes (FDR < 0.01) in MCF-7 cells transfected in triplicate with 10 nM miR-155 or miR-199a5p mimic compared to negative control mimic; top 25 commonly induced genes are listed in heatmap *inset*. (*D*) Culture confluence of MCF-7 cells was monitored using IncuCyte (normalized to initial confluence) over 48 h following transfection of MCF-7 cells with microRNA mimics at 10 nM; SEM of nine scans per well (*n* = 2). Colors as in *A*. (*E*) RT-qPCR analysis of total RNA from MCF-7 cells matched to *D*, collected at 24 h post-transfection. Single gene data is normalized to the geometrical mean of the housekeepers DGUOK and eEF1A1. Error bars. SD of three technical replicates per sample (*n* = 2).

First, we analyzed the effect of miR-155 on MCF-7 growth in 2D culture, using continuous live confluence measurements. Alongside miR-155, we tested a commercial universal negative control, oncogenic miR-21, and five known tumor-suppressor miRNAs natively arising from predominantly two primary transcripts (miR-143/145 and miR-214/199-5p/199-3p, the latter two mature species processed from a common precursor). Figure 1A summarizes the relative observed culture confluence of MCF-7, 48 h after transfection with increasing concentrations of microRNA mimics. Transfecting up to 100 nM of the nontargeting negative control mimic had no observable effects on MCF-7 confluence. For all miRNAs, including the oncogenic miR-21, transfecting increasing concentrations of mimics caused dose-dependent inhibitory effects on MCF-7 growth. Notably, mimic miR-145 caused a substantial reduction in cell confluence even at the lowest concentration tested (5 nM), whereas the mimic of its natively cotranscribed sibling (miR-143) was indistinguishable from the negative control. Strong inhibitory effects were also observed for mimics of miR-155 and miR-199-5p. Interestingly, the ability of miRNA mimics to reduce MCF-7 culture expansion was related to their length, with 21-mer mimics causing no growth phenotype, 22-mer causing a mild inhibition, and 23-mer mimics causing a substantial effect.

To shed light on the mechanisms underlying the growth phenotype, we used microarrays to ascertain changes in gene expression of MCF-7 cells collected 24 h post-transfection with negative control, miR-155 mimics, or miR-199-5p mimics. Differential expression analysis identified a sweeping activation of interferon-related pathways by both 23-mer mimics compared with the negative control (Fig. 1B,C). Strongly up-regulated genes (greater than eightfold) included interferon-β 1 itself (*IFNB1*) as well as the interferon-induced *IFIT1*, *IFIT2*, and downstream oligoadenylate synthases *OAS1*, *OAS2*, and *OAS3*, akin to a Type-I interferon response triggered by double-stranded RNA (dsRNA) intermediates of viral infection (Karpala et al. 2005). Of note, transcripts specifically down-regulated in response to either miRNA mimic were also observed, but being fewer and with substantially smaller fold changes, they were marginalized by the induced interferon response. Figure 1D,E presents a complete time course of MCF-7 growth upon transfection of all miRNA mimics from Figure 1A at 10 nM, and the RT-qPCR analysis of matched samples, validating the dramatic induction (up to 65-fold) of *IFNB1* by all three 23-mer mimics causing growth inhibition.

**FIGURE 2.**
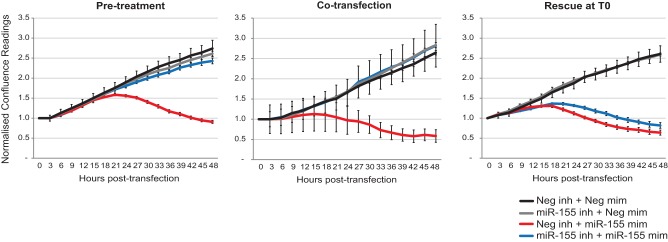
The effect of miR-155 mimic on MCF-7 can be eliminated by antisense inhibitors. Normalized culture confluence (as in Fig. 1) was monitored for 48 h following transfection of MCF-7 cells with combinations of negative control mimic, negative control inhibitor, miR-155 mimic, or miR-155 inhibitor as indicated. (*Left*) Pretreatment with inhibitors 4 h prior to mimic transfection (ratio 20:1); (*middle*) cotransfection of premixed inhibitor and mimic (ratio 5:1); (*right*) transfection of inhibitor and mimic, prepared separately (ratio 5:1). Error bars, SEM of nine scans per well (*n* = 2).

Interferon pathway induction due to the presence of perfect dsRNA in the cytosol has been thoroughly described as a rapid cell response (Karpala et al. 2005). Therefore, we tested whether the effect of miR-155 mimic on MCF-7 cells could be reversed by introduction of an excess antisense miRNA-specific inhibitor. Figure 2 demonstrates the growth of MCF-7 following three schedules of transfection. In cotransfections (middle), transfection of liposomes containing miR-155 mimic premixed with fivefold excess of an antisense inhibitor (but not with the unrelated negative control) abolishes the miR-155-induced reduction in MCF-7 cell density. Thus, only delivery of preformed mimic-inhibitor hybrids evaded perfect dsRNA recognition and deactivated mimic activity. Similarly, pretransfection of the cells with 20-fold excess of inhibitor prior to the transfection of mimic (Fig. 2, left) could also successfully block the growth phenotype, probably because of the immediate and abundant availability of inhibitor in the cytosol. In contrast, concomitant transfection of separately prepared liposomes (Fig. 2, right) of the miRNA mimic and its matched inhibitor failed to alleviate the growth-inhibition phenotype. This suggests that dsRNA recognition is triggered before pairing between mimic and excess inhibitor can occur in the cell, and once the signaling is induced it is irreversible.

**FIGURE 3.**
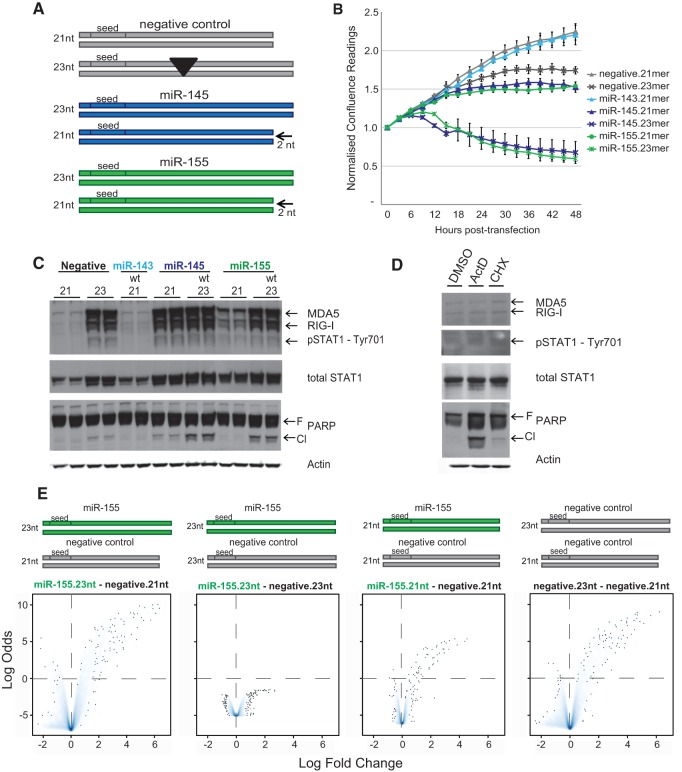
The effect of miR-155 mimic in MCF-7 is length-dependent. (*A*) Schematic illustration of custom lengthened negative control and shortened miR-145 and miR-155 mimics. Not to scale. (*B*) Normalized confluence readings collected post-transfection of MCF-7 cells with 10 nM of mimic variants (as in Fig. 1). Error bars, SEM of nine scans per well (*n* = 2). (*C*) Western blot analysis of MCF-7 cells matched to *B*, collected 24 h post-transfection; protein samples were sequentially probed for the dsRNA-response sensors RIG-I and MDA5, interferon-induced phosphorylated STAT1-Tyr701 and total STAT1, apoptosis marker PARP (F: full-length; Cl: cleaved), and actin as a loading control. (*D*) Western blot analysis of dsRNA-response elements (as in *C*) in MCF-7 cells collected 24 h post-treatment with apoptosis inducing actinomycin D (10 nM), cyclohexamide (100 nM), or DMSO alone. (*E*) Volcano density plots (as in Fig. 1) of microarray-based differentially expressed genes of MCF-7 cells transfected the indicated mimic variants. The *y-*axes are on a common scale.

To establish whether the concomitant growth-inhibition phenotype and induction of the interferon-β pathway were indeed associated with the differential length of the miRNA mimics, we collaborated with the manufacturer (QIAGEN) to generate variant miRNA mimics outlined in Figure 3A: an extended 23-mer negative control variant (bearing a 2-nt insertion in its middle to avoid affecting the seed sequence on either RNA strand) and shortened 21-mer variants of miR-155 and miR-145 mimics (2 nt truncated at 3′ end of the native sequence, commonly believed to be of little significance in seed-driven RISC-mediated functions of miRNAs).

Following transfection of these mimic variants, we observed the growth of MCF-7s (Fig. 3B) and assessed the levels of key proteins in dsRNA signaling and apoptosis (Fig. 3C)—namely, the dsRNA sensors RIG-I and MDA5, total and Tyr701-phosphorylated interferon-induced STAT1, and the cleavage of PARP as a marker for apoptosis. For both miR-155 and miR-145, shortening of the mimic substantially alleviated MCF-7 growth suppression compared to the full-length variants consistently with a reduction in cleaved PARP. Conversely, lengthening of the negative control caused a reduction in culture growth and triggered dsRNA recognition and signaling, albeit to a lesser extent than any of the miR-155/145 variants. Transfected miR-143, the native cotranscribed sibling to miR-145, had no effect on MCF-7 cells (see also Fig. 1). A similarly designed shortening of miR-199a5p to a 21-mer variant entirely eliminated the MCF-7 growth phenotype induced by its 23-mer (data not shown). Importantly, induction of apoptosis in MCF-7 cells using actinomycin D or cyclohexamide does not affect dsRNA sensors or STAT1 (Fig. 3D), confirming the unidirectionality of the “dsRNA mimic → interferon response → apoptosis” chain of events.

We then performed a microarray gene expression analysis of MCF-7 cells 24 h post-transfection with miR-155 or negative control variants. Figure 3E presents volcano plots of selected comparisons. In particular, the comparison between 21-mer and 23-mer variants of the negative control (far right) highlights the magnitude of dsRNA signaling (up to 64-fold induction; similar to the original miR-155 23-mer vs. 21-mer negative contrast on the far left). Moreover, comparing miR-155 and negative control mimics of similar lengths (middle left, 23-mers; middle right, 21-mers) demonstrates how potentially physiologically relevant miRNA-specific effects observable using 21-mer mimics lose any significance in the context of a transcriptionally dominant dsRNA cell response when used as 23-mers.

**FIGURE 4.**
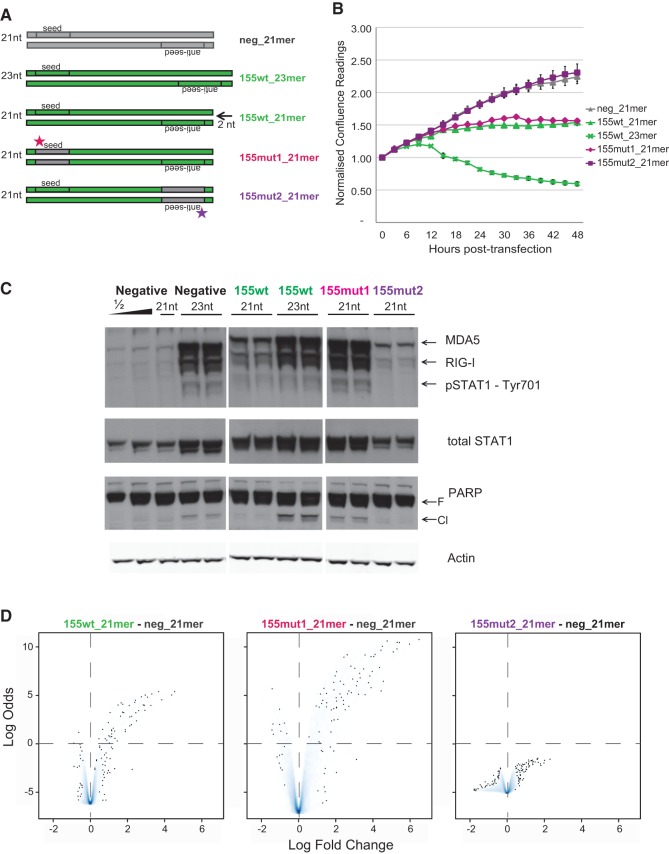
The effect of miR-155 mimic in MCF-7 depends on the seed of the passenger strand. (*A*) Schematic illustration of standard negative control and miR-155 mimics and custom shortened and/or mutated variants of miR-155 (21-mer wild-type, mutant 1 and mutant 2). Anti-seed, passenger strand seed sequence. Not to scale. (*B*) Normalized confluence readings collected post-transfection of MCF-7 cells with 10 nM of mimic variants (as in Fig. 1). Error bars: SEM of nine scans per well (*n* = 2). (*C*) Western blot analysis of MCF-7 cells matched to *B*, collected 24 h post-transfection; protein samples were sequentially probed as in Figure 3. Lanes of negative control and miR-155 wt mimics are repeated from Figure 3 for comparison. (*D*) Volcano density plot (as in Fig. 1) of microarray-based differentially expressed genes of MCF-7 cells transfected the indicated mimic variants. The *y*-axes are on a common scale.

In light of the known functions miR-155 exerts in the immune system (Vigorito et al. 2013), we next turned to investigate the residual induction of interferon signaling by the 21-mer variant of miR-155 (Fig. 4). To examine whether it was physiologically relevant and dependent on the miRNA seed sequence, we generated two further 21-mer mimic variants. In the first variant (miR-155mut1), miR-155's native seed sequence was substituted by the nontargeting seed of the commercial negative control. In the second variant (miR-155mut2), the same nontargeting seed sequence was introduced into the passenger strand. The resulting variants are schematically outlined in Figure 4A. The seed-altered miR-155mut1 was as potent as its wild-type counterpart (miR-155wt 21mer) in repressing MCF-7 growth, suggesting that the phenotype was not driven by the seed sequence (Fig. 4B). Interestingly, miR-155mut1 was even more efficient at inducing dsRNA signaling than the 21-mer miR-155wt, although not to the extent observed with the 23-mer variant (Fig. 4C). Unexpectedly, the MCF-7 growth response to miR-155mut2 transfection was indistinguishable from the negative control. Although dsRNA sensors were mildly up-regulated compared to control, this was not sufficient to induce total or phosphorylated STAT1 protein levels nor apoptosis. Thus, the growth suppression observed in Figure 1 by 23-mer miR-155 mimic seems to be a compound effect of its length and the seed-dependent activity of its artificial passenger strand.

**FIGURE 5.**
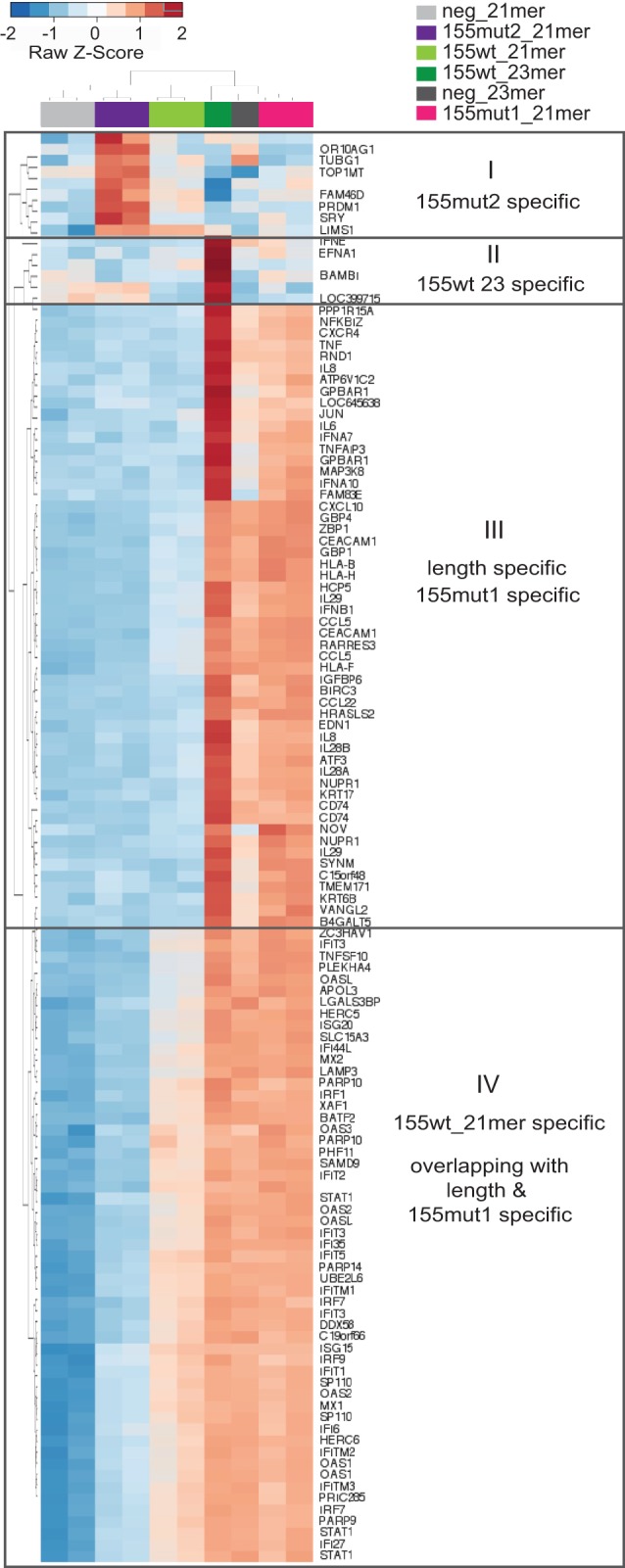
Variant-specific changes in gene expression. The heatmap depicts unsupervised clustering of the union of the top 50 most deregulated Illumina probes in each of the six contrasts (155wt_21mer versus neg_21mer; 155mut1_21mer versus neg_21mer; 155mut2_21mer versus neg_21mer; 155wt_23mer versus neg_21mer; neg_23mer versus neg_21mer; 155wt_23mer versus neg_23mer). Mimics are color-coded as in Figure 4.

Microarray profiling, illustrated in Figure 4D, was used to ascertain the transcriptome differences induced by mimic transfections matched to Figure 4B. Primarily, the “passenger seed” mutant, miR-155mut2, showed marginal differential expression compared to the negative control (right), concurring with the absence of induced growth phenotype in cell culture. Contrastingly, miR-155mut1 (middle) caused more numerous and substantial fold changes in gene expression than the 21-mer wild-type mimic (left), akin to the effect of the original 23-mer variant. Indeed, analysis of the combined top 50 most up-regulated genes across six comparisons of the 21-mer variant mimics (Fig. 5) reiterated components of the interferon pathway as the key differentially expressed genes. Both 21-mer miR-155wt and miR-155mut1 mimics induced the expression of *STAT1*, *IFITs*, *IRFs*, and *OASs* among others, similarly to the 23-mer miR-155wt but also the 23-mer negative control. However, the miR-155wt 21-mer triggered only a mild up-regulation compared with the 23-mers and miR-155mut1, which additionally induced tertiary targets like secreted cytokines (*ILs*, *CCLs*, *CXCL10*), receptors, and antigen presenting molecules (*CXCR4*, *CEACAM1*, *CD74*). Up-regulation of tertiary targets was proportional to the extent of observed growth retardation, suggesting that their activation contributes to the growth retardation phenotype. In contrast, genes down-regulated by mimic transfection were not consistent between mimics bearing identical seeds (data not shown). This is probably a result of the low number of such targets and smaller expression fold changes, jointly resulting in a weaker and less consistent contribution to the differential expression analysis—and probably to the cellular phenotype.

**FIGURE 6.**
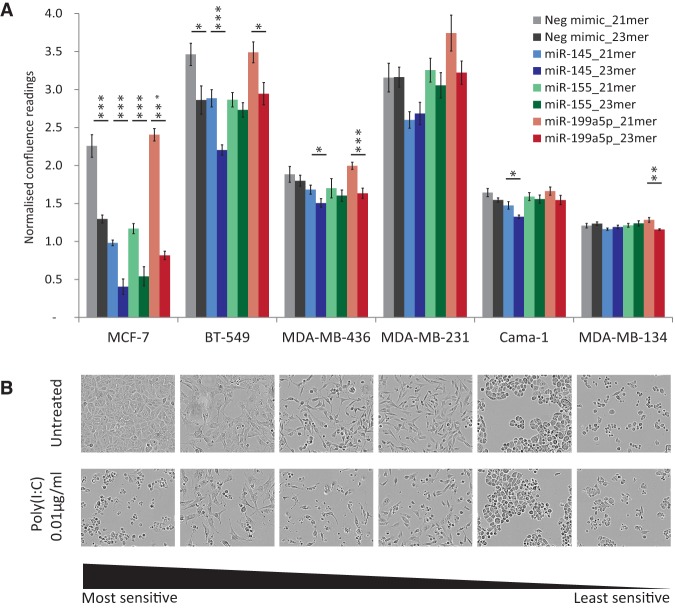
Sensitivity to miRNA mimic length is cell type–specific. (*A*) Culture confluence (normalized to initial confluence) of the indicated six breast cancer cell lines was measured using an IncuCyte at 48 h post-transfection of 10 nM mimic variants (as in Fig. 1). Error bars, SEM of nine scans; D'Agostino and Pearson normality test to identify parametric data; unpaired student *t*-test: (*) *P* < 0.05; (**) *P* < 0.01; (***) *P* < 0.001. (*B*) Light microscopy images taken 48 h post transfection of dsRNA Poly (I:C) or matched untreated cultures of the same six cell lines. Cell lines are ordered from most to least sensitive to Poly (I:C) transfection.

Lastly, to determine if the sensitivity to 23-mer mimics and subsequent induction of dsRNA signaling was unique to MCF-7 cells, transfections of the 21-mer and 23-mer mimic variants were repeated in five additional breast cancer cell lines (Fig. 6A). There was a continuous range of length-dependent and miRNA-dependent cell responses, with MCF-7 marking the extreme sensitive end of the spectrum and both Cama-1 and MDA-MB-134 cells showing hardly any response to 23-mers. MDA-MB-231 cells appeared to show some cell growth sensitivity to miR-199 23-mer; however, this was not found to be statistically significant (*P*-value = 0.08). The same graduated sensitivity of the cell lines to dsRNA was also observed when transfecting a traditional nonspecific long dsRNA Poly (I:C) (polyinosinic–polycytidylic acid [Wang et al. 2015]), in agreement with a common mechanism (Fig. 6B).

## DISCUSSION

The recognition of miRNAs as key players and modulators in normal and pathological processes has spurred a flurry of research, much of which revolves around cellular responses to perturbation of individual miRNA levels. This is commonly achieved by transfection of synthetic mimics or inhibitors. We show that a miR-155 mimic causes a growth-retardant and apoptosis-inducing effect in MCF-7 cells, associated with the induction of a dsRNA response and the interferon pathway. Using a series of custom variants, we clarify that these effects are mostly driven by the length of the mimic and its fully complementary passenger strand. The effects can be reduced by shortening the mimic and completely abolished by mutating the passenger seed sequence. Thus, it appears that the immune-related interferon activation by miR-155 mimic is artifactual and unrelated to the miR-155's native function in the immune system. Indeed, a miR-145 mimic elicits a similar phenotype, although miR-145 is not generally known for its involvement in the immune response and has even been reported to repress *IFNB* in macrophages (Witwer et al. 2010).

Commercial negative controls (with “scrambled control” being a frequently used misnomer throughout the literature) are predominantly a legacy from R&D of siRNA, which are by-and-large 21-mers. As such, they do not provide an adequate match for longer miRNAs to control for length-specific induction of dsRNA response. This drawback cannot be dismissed as a rare anecdote because miRNAs of 23 nt or longer comprise 20.1% of known human miRNAs (24.4% across all species; miRBase21 [Kozomara and Griffiths-Jones 2014]). Although the list of miRNAs of 24 nt or longer is dominated by high miR indices referring to unconfirmed or poorly studied miRNAs, the 23-nt list includes well-studied miRNAs like miR-10a-5p, the miR-181-5p family, and members of the multifunctional miR-17-92 “oncomiR-1” polycistron (Olive et al. 2013). Furthermore, we show that cell lines exhibit differential sensitivity to mimic length, complicating the interpretation of context-specific responses, such as the reported contradictory activities of the 24-nt miR-125a-5p (for review, see Sun et al. 2013) or the cell type–specific induction of apoptosis by the 23-nt miR-145 (Spizzo et al. 2010), also evident in our work.

It is clear from our data that length alone does not account for the extent of dsRNA response, so that a 23-mer negative control provokes a milder response than the native miR-155 and -145 mimics, whereas the 21-mer miR-155 mutants 1 and 2 display a distinctly different interferon-inducing activity. Whether this difference can be attributed to genuine sequence-specific effects, the sequence's GC content, interaction with cellular RNA binding proteins, or additional parameters is at present unknown.

Our findings regarding length-dependent activation of interferon response by short duplexes are supported by similar published observations regarding siRNA reagents (Reynolds et al. 2006; Ishibashi et al. 2011), while passenger-strand effects were recently investigated in silico by Søkilde et al. (2015). Despite reports that siRNA-induced gene silencing can be activated by duplexes as short as 16 nt (Johansson et al. 2013), the siRNA field converged on an industry standard of 21-mer reagents. In our hands 21-mers indeed appear to be free from confounding interferon-induced changes to gene expression and possibly cell fate in most, but not all, cell lines. It is imperative that the miRNA community reaches similar rigorously tested standards—in particular, if miRNA mimics are to be introduced as therapeutic reagents across multiple clinical disciplines (van Rooij and Kauppinen 2014). Various improvements have been recently suggested by several laboratories and manufacturers in the form of modifications to the chemistry of the passenger strand of miRNA mimics creating non-dsRNA alternatives that could circumvent dsRNA response or the use of single-stranded reagents (Peacock et al. 2011; Chorn et al. 2012) or fine-tuning the termini and thermodynamic features of mimics (Hu et al. 2009; Chang et al. 2013) to enhance the incorporation of the sense strand into RISC and reduce passenger-driven artifacts. However, the need for length-matched negative controls remains even for those reagents. Ultimately, miRNA-specific seed mutants and orthogonal experimental approaches are the correct way to control for the specificity of an observed phenotype despite adding to the cost and complexity of the experimental design.

## MATERIALS AND METHODS

### Cell culture, transfections, and density readings

Breast cancer cell lines were manipulated under sterile conditions in a Class II laminar flow hood, maintained in 37°C incubators (5% CO_2_, 5% O_2_) and passaged 1:2-1:6 by trypsinization at ∼90 % confluence. Where necessary, cell density and viability of suspensions were assessed by Vi-CELL Analyzer (Beckman Coulter). Cell aliquots were regularly tested for mycoplasma contamination using the RNA-capture ELISA-based MycoProbe Kit (R&D Systems). Media and supplements (Invitrogen) used for cell line propagation and the cells’ estrogen receptor (ER) status are listed below. DMEM-GlutaMAX + 10% FBS used for Cama-1 (ER+), MCF-7 (ER+), MDA-MB-134 (ER+), and MDA-MB-231 (ER−); RPMI-1640 + 10% FBS used for MDA-MB-436 (ER−); RPMI-1640 + 10% FBS + 0.023I U/mL insulin (Sigma-Aldrich) used for BT-549 (ER−).

Polyinosinic–polycytidylic acid sodium salt [Poly (I:C)] was purchased from Sigma-Aldrich. miScript miRNA mimics and modified single-stranded RNA inhibitors purchased from QIAGEN are listed below. Sequences of wild-type miRNAs with matched custom variants are provided for reference: miR-1 (MSY0000416), miR-21 (MSY0000076), miR-143 (MSY0000435), miR-145 (MSY0000437: GUCCAGUUUUCCCAGGAAUCCCU), miR-155 (MSY0000646: UUAAUGCUAAUCGUGAUAGGGGU), miR-183 (MSY0000261), miR-199a3p (MSY0000232), miR-199a5p (MSY0000231: CCCAGUGUUCAGACUACCUGUUC), miR-214-3p (MSY0000271) and the AllStars Negative control (1027281; XXXXXXXXXXXXXXXXXXXXX), miR-155 inhibitor (MIN0000646) and the miScript inhibitor negative control (1027272; proprietary sequence). Custom mimic variants, HPLC-purified miR-155 mimic, and seed mutants were generated and provided by QIAGEN on a collaborative basis (miR-145 21-mer: GUCCAGUUUUCCCAGGAAUCC; miR-155 21-mer: UUAAUGCUAAUCGUGAUAGGG; miR-155mut1: UXXXXXCUAAUCGUGAUAGGG; miR-155mut2 [passenger strand listed]: CXXXXXCACGAUUAGCAUUAA; miR-199a5p 21-mer: CCCAGUGUUCAGACUACCUGU; negative control 23-mer: XXXXXXXXXXXAAXXXXXXXXXX). Complete sequences of the four reagents bearing the negative control seed (underlined) are proprietary and are available upon request subject to a confidentiality agreement with QIAGEN.

Transfections were typically performed in 24-well plates on subconfluent cultures of cell lines using 2–200 nM reagents as indicated for each experiment, using Lipofectamine 2000 transfection reagent (Invitrogen) following manufacturer's instructions. Consistent transfection efficiency across the different cell lines was monitored by RT-qPCR or fluorescein-labeled negative control reagents (QIAGEN AllStars negative control siRNA 1027282). Transfected cells were either maintained in standard incubators for 24–48 h prior to RNA and protein extraction or transferred immediately into the Incucyte Live Cell Imaging System (Essen Bioscience) for confluence reading analysis. In brief, phase-contrast images were automatically collected at nine positions in each well every 3 h over the course of 2–7 d as indicated. The resulting measurements of the total surface area focused on the plane of adherent cells (representing % confluence or density) was normalized to the initial confluence for each sample to account for differential seeding density.

### RNA extraction and gene expression analysis

Cells of 24–48 h post-transfection were washed with 1× PBS and lysed in QIAzol (QIAGEN). Total RNA was extracted using the miRNeasy Mini Kit (QIAGEN) according to the manufacturer's instructions. RNA yield and purity was assessed by Nanodrop (Thermo Scientific). Microarray analysis was performed using Illumina Beadarrays (Human v4 Beadchip) and processed for analysis as in Curtis et al. (2012). MetaCore (GeneGO, Thomson Reuters) software was utilized for pathway enrichment analysis of gene lists generated from up- or down-regulated differentially expressed genes.

Genome-wide measurement of changes to gene expression was carried out using Illumina BeadArrays and processed as in Curtis et al. (2012). Normalized summarized data are available through the Gene Expression Omnibus (GEO) archive under accession number GSE75802.

Measurement of individual transcripts was performed by reverse-transcription (RT)-real time PCR (qPCR). To allow measurement of both mRNA and miRNA levels from single reverse-transcription reactions, RNA was first polyadenylated using the poly(A) polymerase (PAP) kit (Ambion) (Git et al. 2008). In brief, 300 ng total RNA was vacuum dried and resuspended in 0.8 µL 5× PAP buffer, 0.4 µL 25 mM MnCl_2_, 0.4 µL 10 mM dATP, 0.08 µL poly(A) polymerase, and 2.32 µL of RNase-free water. Reactions were incubated at 37°C for 1 h and inactivated at 70°C for 15 min. Polyadenylated RNA was reverse transcribed by Superscript III Reverse Transcriptase (Life Sciences) primed by 270 ng of random hexamers and 140 ng of oligo(dT)-anchor primer (GCGAGCACAGAATTAATACGACTCACTATAGGTTTTTTTTTTTTVN) at 25°C for 5 min, at 55°C for 1 h and then inactivated at 70°C for 15 min. RNA was then digested with 2 uL RNase H (Invitrogen) for 30 min at 37°C and diluted 1:100.

To measure mRNA or miRNA expression, triplicate 5 μL aliquots were subjected to real-time PCR in 1× FAST SYBR Green Mastermix (Applied Biosystems) following the recommended cycling program on a 7900HT instrument (Applied Biosystems) primed by 500 nM of the intron-spanning exonic primer pairs (DGUOK GCTGGTGTTGGATGTCAATG and GCCTGAACTTCATGGTATTGG; eEF1A1 GGCATCGACAAAAGAACCAT and CCCAGGCATACTTGAAGGAG; IFNB1 GTCACTGTGCCTGGACCATAG and GCTAGGAGATCTTCAGTTTCGG). miRNA mimic presence in the cells (data not shown) was measured by priming with a universal reverse anchor primer (GCGAGCACAGAATTAATACGACTC) and miRNA-specific forward primers (miR-193 CAAAGTGCTGTTCGTGCAGGTAG; miR-145 GTCCAGTTTTCCCAGGAATCCCTT; miR-155-5p TTAATGCTAATCGTGATAGGGGT; miR-191 CAACGGAATCCCAAAAGCAGCTG; miR-199a-5p CCCAGTGTTCAGACTACCTGTTC; miR-199a3p TACAGTAGTCTGCACATTGGTT). In compliance with miQE guidelines (Bustin et al. 2009), a representative pooled sample was used for a series of additional controls: 600, 300, and 150 ng of input RNA to demonstrate a dynamic response of the reverse transcription; a serial dilution curve of a single reverse-transcription reaction for accurate determination of relative quantities where 1 unit Ct did not correspond to a twofold increase in input; a PAP-free reaction to confirm a nonpolyadenylated source for miRNA amplification; an RT-free reaction to account for RNA-independent nonspecific amplification; a template-free reverse-transcription; and a template-free PCR to ensure no reagent contamination and dominant primer dimers. End products were analyzed by an automated thermal dissociation curve to assure a single amplified product. Relative expression of genes of interest was calculated based on the titration curve and normalized to the geometric mean of eEF1A1 and DGUOK, the most stably expressed microarray transcripts across the METABRIC cohort of breast tumors (Curtis et al. 2012).

### Protein extraction and Western blot analysis

In brief, 24–48 h post-transfection cells were washed with 1× PBS and resuspended in 100–150 µL protein lysis buffer (50 mM Tris–HCl pH 7.5, 5 mM EDTA, 150 mM NaCl, 1% v/v Triton X-100, 50 mM NaF, 25 mM β-glycerophosphate, 1 mM sodium orthovanadate, 1× complete EDTA protease inhibitors [Roche]). Protein concentration was determined by a BCA assay (Pierce) relative to BSA standards. Ten or 20 µg lysate were separated on SDS-PAGE gels (cast at 12% resolving, 8% stacking), alongside a protein marker mixture (1:1 Benchmark prestained protein ladder [Life Technologies] and MagicMarkXP standard [Life Technologies]), transferred onto a nitrocellulose membrane using the iBlot Dry Blotting System (Life Technologies) and subjected to Western blot analysis. The primary antibodies used were 1:10,000 β-Actin (Abcam ab6276); 1:1000 Caspase-3 (Cell Signaling 9665); 1:1000 MDA5 (Cell Signaling 5321); 1:500 PARP (Cell Signaling 9542); 1:1000 RIG-1 (Cell Signaling 3743); 1:1000 total-STAT1 (Cell Signaling 9172); and 1:1000 phospho-STAT1-Tyr701 (Cell Signaling 9171). Secondary antibodies were HRP-conjugated Goat anti-rabbit (Dako P0448) or Goat anti-mouse (Dako P0447) and were used at 1:2000 in 5% BSA, TBST (0.1% v/v Tween, 150 mM NaCl, 20 mM Tris–HCl pH 7.6). HRP was visualized following 1 min incubation in 1:1 mixture of homemade ECL Solution 1 (0.25 mM Luminol, 0.37 mM P.Coumaric acid, 0.1 M Tris–HCl pH 8.5) and Solution 2 (0.018% H_2_O_2_, 0.1 M Tris–HCl pH 8.5) and exposed on X-ray film. Serial probing for proteins of distinct molecular weights was performed without stripping of membranes. Ten and 20 µg of a positive lysate were included alongside experimental sample to ensure nonsaturating semiquantitative conditions.
